# A plant biostimulant made from the marine brown algae *Ascophyllum nodosum* and chitosan reduce *Fusarium* head blight and mycotoxin contamination in wheat

**DOI:** 10.1371/journal.pone.0220562

**Published:** 2019-09-11

**Authors:** L. R. Gunupuru, J. S. Patel, M. W. Sumarah, J. B. Renaud, E. G. Mantin, B. Prithiviraj

**Affiliations:** 1 Department of Plant, Food and Environmental Sciences, Faculty of Agriculture, Dalhousie University, Truro, Nova Scotia, Canada; 2 Agriculture and Agri-Food Canada, London, Ontario, Canada; Institute of Genetics and Developmental Biology Chinese Academy of Sciences, CHINA

## Abstract

*Fusarium* head blight (FHB) caused by *Fusarium graminearum* is a disease that results in yield loss and mycotoxin contamination in wheat globally. This study assessed the effect of a plant biostimulant prepared from a brown macroalga *Ascophyllum nodosum* (Liquid Seaweed Extract; LSE) alone and in combination with chitosan in controlling *Fusarium*. Wheat seedlings drenched with LSE and chitosan in combination showed reduced severity of *F*. *graminearum* infection on leaves as evidenced by a significant reduction in necrotic area and fewer number of conidia produced in the necrotic area. Gene expression studies showed that the combination of LSE and chitosan amplified the response of pathogenesis-related genes (*TaPR1*.*1*, *TaPR2*, *TaPR3*, *TaGlu2*) in wheat seedlings infected with *Fusarium* spores above that observed for the individual treatments. The combination treatments were more effective in enhancing the activity of various defense related enzymes such as peroxidase and polyphenol oxidase. FHB studies on adult plants showed a reduction of bleached spikes in wheat heads treated with the combination of LSE and chitosan. Mycotoxin content appeared to be correlated with FHB severity. Combination treatments of LSE and chitosan reduced the levels of mycotoxins deoxynivalenol and sambucinol in wheat grains. Systemic disease resistance appears to be induced by LSE and chitosan in response to *F*. *graminearum* in wheat by inducing defense genes and enzymes.

## Introduction

Plants possess inducible defense mechanisms, utilizing effector molecules to defend against pathogens [1]. Plant defense responses are induced by the application of elicitor molecules in two ways: locally or systemically. These elicitor molecules are isolated from a number of sources including plants, animals, cell wall components of microbes and cellular components of avirulent pathogens [2,3]. Seaweeds are a rich source of bioactive compounds that reportedly enhance plant productivity and improve overall plant health. Many studies suggest that seaweed extracts benefit plants by inducing early seed germination, improving overall growth and enhancing tolerance to biotic and abiotic stresses. Seaweed polysaccharides and their derivatives induce plant defense responses and protect plants from a wide range of pathogens by activating defense pathways (salicylic acid, jasmonic acid and ethylene) [4–6]. The induction of defense hormone pathway genes will in turn activate various genes that encode pathogenesis-related proteins in plants [7]. Elicitor molecules like oligosaccharides and glycol peptides activate various defense responses and prime plants for tolerance and or resistance against both biotic and abiotic stresses.

Chitosan (de-acetylated chitin) derivatives isolated from fungi or crustaceans are biologically active and induce plant tolerance or resistance against a wide range of pathogens in conjunction with possessing direct antifungal activity against certain fungi [8,9]. In agriculture, chitosan can be used as both a soil amendment and foliar spray against pathogens [10–12]. Wheat seeds and seedlings treated with chitosan showed enhanced resistance to seedling blight and head blight caused by *Fusarium graminearum* [12,13].

*Fusarium* spp. including *F*. *culmorum* and *F*. *graminearum* cause seedling blight, root rot and head blight diseases in wheat and cause major economic losses to farmers. Fusarium head blight (FHB) infection also results in mycotoxin contamination of grains [14,15]. With the risk of mycotoxin contamination of grains and the lack of FHB resistance in commercial cultivars of wheat, the control of FHB has received significant attention. FHB control in wheat through the application of naturally available compounds is gaining momentum due to increasing restrictions on the use of synthetic fungicides. In the present study, we tested the efficacy of a plant biostimulant formulation made from a brown alga, *Ascophyllum nodosum*, alone and in combination with chitosan on *Fusarium* infection and on mycotoxins. The mode of action of the seaweed extract formulation was also included in this study.

## Materials and methods

### Plant and fungal culture

The wheat (*Triticum aestivum*) cultivar ‘Helios’ used in this experiment was provided by Michael Main (Faculty of Agriculture, Dalhousie University, NS, Canada). ‘Helios’ has an intermediate resistance to FHB disease [16]. For greenhouse experiments, wheat seeds were germinated in darkness for 72 hours at 24°C in Petri dishes containing moist Whatman No. 1 filter paper, as per the germination protocol described by Gunupuru et al. [15]. Germinated seedlings were transferred into 3 L plastic pots containing PRO-MIX **(**Premier Tech Horticulture, QC, Canada) and were grown under greenhouse conditions with a day/night temperature regime of 24/18°C and light/dark regime of 16/8 hours [15]. Wild-type *F*. *graminearum* strain DAOM180378, isolated in Ottawa, ON, Canada was obtained for this research from the Canadian Collection of Fungal Cultures (CCFC) [17]. This strain was isolated from maize in Ottawa, ON, Canada in 1981 by G.A. Neish and was used in this research for its abilities to infect wheat and produce the mycotoxin deoxynivalenol [18,19]. An inoculum of *F*. *graminearum* spores (asexual) (10^6^ conidia mL^-1^) was prepared following method described earlier [20] [21].

### Plant biostimulant (LSE) and chitosan preparation

The plant biostimulant made from *A*. *nodosum* (Liquid Seaweed extract, LSE) was a gift from Acadian Seaplants Limited (Dartmouth, NS, Canada). A 2000 ppm stock solution of crab shell chitosan (85% deacetylated, Sigma-Aldrich) was prepared in 0.5 M aqueous acetic acid following the protocol of Khan et al. [12]. The solution was adjusted to pH 5.2 with 1 N KOH and autoclaved at 121°C for 15 minutes [12]. Acetic acid (0.5 M, pH 5.2) served as a control for chitosan treatments [12]. Experimental treatments consisted of LSE and chitosan, individually and in combination (Table 1).

**Table 1:** Treatment abbreviations and formulations (individual and in combination) used throughout experiments.

Treatment	Concentration of *A*. *nodosum* extract (LSE) (mL L^-1^)	Concentration of Chitosan (ppm)	Other
**T1**	-	-	0.02% Tween20 (Control)
**T2**	-	100	-
**T3**	5	-	-
**T4**	15	-	-
**T5**	5	100	-
**T6**	15	100	-

### Leaf detachment assay

Twenty-day old wheat seedlings were drenched with 5 mL of treatment solution. Forty-eight hours post-treatment, the second leaf was removed and used to conduct a detached leaf disease assay, modified from Browne and Cooke [22]. A leaf section of the second leaf from a three-leaf-stage plant was cut (8 cm) and placed with the cut end between two layers of agar containing 0.5 mM benzimidazole (1%, pH 5.7, BioShop) [23]. This leaf section was placed in a Petri dish (90 mm square plate) with the adaxial surface facing upward [22,23]. Each leaf section was punctured at the centre and subsequently treated with a 4 μL of either a control solution (0.02% (v/v) Tween20) or the *F*. *graminearum* (10^6^ conidia mL^-1^) [22,23]. Petri dishes were incubated at 20°C under a light/ dark regime of 16/8 hours for three days. Diseased leaf area was estimated three days post-inoculation by taking photos of the leaf sections and measuring the area using ImageJ software [23,24]. Macroconidia produced on the leaf sections were measured using a haemocytometer (HYCOR Biomedical) after being rinsed with water and vortexed [23]. There were three biologicals replicates per treatment and three leaf section replicates.

### *In vitro* antifungal assay

Potato dextrose agar (PDA) plates were prepared then amended with different concentrations of autoclaved LSE and chitosan, individually and in combination (concentrations from Table 1). A 5 mm mycelial plug of one-week old *F*. *graminearum* culture was placed at the centre of each PDA plate and incubated at 25°C for five days in darkness. After incubation, photographs that included a reference ruler were taken and analysed using ImageJ software. The percentage of fungal growth inhibition was calculated through the following formula:

$$\%Inhibition=\left(\frac{radial\;mycelial\;growth\;(control\;plate)-radial\;mycelial\;growth\;(treatment\;plate)}{radial\;mycelial\;growth\;(control\;plate)}\right)\times 100$$ (1)

### The effect of LSE and Chitosan on FHB disease severity, defense enzymes, gene expression and mycotoxin contamination

#### Plant culture, LSE and chitosan treatment and pathogen inoculation

Wheat plants were grown in the greenhouse as described previously: seeds were germinated in darkness for 72 hours in Petri dishes on moist Whatman No. 1 filter paper at 24°C. Germinated seedlings were transferred into 3 L pots containing PRO-MIX and were grown under a day/ night temperature regime of 24/18°C and light/ dark regime of 16/8 hours [15]. Two-leaf stage wheat seedlings were root drenched with 5 mL of treatment solutions noted in Table 1 (twelve seedlings per pot, 5 pots per replicate, 3 replicates per treatment). Forty-eight hours post-drenching, each seedling was sprayed with 1 mL of freshly isolated *F*. *graminearum* spores suspended in 0.02% Tween20 at a concentration of 4 × 10^4^ conidia mL^-1^ (control plants were sprayed with 1 mL of 0.02% Tween20). The pots were covered with transparent domes to maintain humidity. Three seedlings per pot were collected and pooled per time point at 0, 24, 48- and 72-hours post-inoculation and immediately stored in liquid nitrogen for gene expression analysis and biochemical quantification.

#### Phenylalanine ammonia lyase (PAL) assay

Enzyme extraction was carried out using a modified protocol by Aydaş et al. [25]. A leaf sample (~ 1 g) previously collected from three wheat seedlings at four time points, as described in the previous section, was pulled from frozen storage and crushed in 5 mL of 500 μmol Tris HCl buffer (pH 7.0, 4°C) and centrifuged at 12,000 g for 15 minutes at 4°C. The resultant supernatant was collected and used as the extracted enzyme from the plant. Following collection, the plant extract supernatant (200 μL) was mixed with 400 μL of reaction buffer (100 mmol/L potassium phosphate, pH 7) and 200 μL of substrate (40 mmol L^-1^ of L-phenylalanine, 100 mmol L^-1^ potassium phosphate, pH 7) and incubated at 37°C for 15 minutes [25]. An aliquot (200 μL) of 250 g L^-1^ trichloroacetic acid (TCA) was added to terminate the reaction, followed by centrifugation at 13,000 g for 15 minutes [25]. Trans-cinnamic acid content was determined using the optical density (OD) at 290 nm against a standard curve [25].

#### Polyphenol-oxidase (PPO) assay

Polyphenol-oxidase (PPO) activity was determined following the protocol of da Silva and Koblitz[26]. A wheat seedling leaf sample (taken from frozen storage) was homogenised in 0.1 M phosphate buffer (pH 7.0, 4°C) and centrifuged at 12,000 g for 15 minutes at 4°C. The resultant supernatant (100 μL) was mixed with 2.3 mL phosphate buffer (50 mM, pH 7.0) and 0.6 mL pyrocatechol solution (100 mM) and the solution was maintained in a water bath at 25°C [26]. The optical density at 425 nm was measured 10 minutes after the addition of the enzyme extract (supernatant). A control solution without the enzyme extract was used as a blank.

The following was used to calculate oxidase activity of the enzyme extract [26]:

$$Activity\;(U\;{mL}^{-1})=\frac{\left[\left({AF}_{SAMPLE}-{AI}_{SAMPLE})-({AF}_{BLANK}-{AI}_{BLANK}\right)\right]}{0.001\times t}$$ (2)

AF_sample_ and AI_sample_ are the final and initial absorption values of the sample, respectively and AF_blank_ and AI_blank_ are the final and initial absorption values of the control, respectively. The reaction time is represented by ‘t’ [26].

#### Peroxidase (PO) assay

Peroxidase activity was determined following the protocol described by Aydaş et al. [25]. One gram of a wheat seedling leaf sample was homogenised in 4 mL of 0.1 M phosphate buffer (pH 7.0, 4°C) and centrifuged at 12,000 g for 15 minutes at 4°C [25]. The resultant supernatant was collected and used as the extracted enzyme from the plant. The reaction mixture contained 0.1 mL enzyme extract, 2.8 mL of 0.1 M phosphate buffer (pH 7), 0.05 mL of 20 mM guaiacol and 0.1 M H_2_O_2_ [25]. Changes in OD were measured immediately at 436 nm at 50 second intervals for 3 minutes, a modified measurement protocol from Pütter [27]. The enzyme activity was expressed as the change in OD per minute per gram of fresh weight [25].

#### Gene expression analysis using quantitative reverse transcriptase PCR analysis

Total RNA from leaf tissue was extracted using a GeneJET^™^ Plant RNA Purification Kit (Thermo Scientific^™^) following the manufacturer’s instructions. DNase treatment of RNA was performed using DNase 1 solution (ThermoFisher^™^). The concentration of RNA was measured using a NanoDrop^™^ 1000 (Thermo Scientific^™^) and the integrity was visualized on a agarose gel after electrophoresis [23]. Reverse transcription of total RNA was performed as described previously [28] using a cDNA Reverse Transcription Kit (Applied Biosystems). The reaction mixture (15 μL containing 2 ng of cDNA synthesized with 300 nM of gene specific primers (Invitrogen^™^) (Table 2) and 7.5 μL SYBR Green master mix (BioRad) was loaded onto qPCR reaction plates (Applied Biosystems). The reactions were run on a StepOnePlus Real-Time PCR system according to Applied Biosystems protocols. Using the comparative C_T_ method (ΔΔC_T_) method with GAPDH as a control, the relative expression of four genes of interest (*TaPR1*, *TaPR2*, *TaPR3* (Chitinase) and *TaGlu2*) were calculated [28,29].

**Table 2:** Gene specific primers (Invitrogen^™^) used for real time reverse transcription polymerase chain reaction (RT-PCR) to quantify the gene expression of four genes of interest (*TaPR1*, *TaPR2*, *TaPR3* and *TaGlu2*) in treated wheat seedlings.

Primer Name	Sequence (5’– 3’)
TaPR1/1:FP	CTGGAGCACGAAGCTGCAG
TaPR1.1:RP	CGAGTGCTGGAGCTTGCAGT
TaPR2:FP	CTCGACATCGGTAACGACCAG
TaPR2:RP	GCGGCGATGTACTTGATGTTC
TaChitinase(PR3):FP	AGAGATAAGCAAGGCCACGTC
TaChitinase(PR3):RP	GGTTGCTCACCAGGTCCTTC
TaGlu2:FP	CCAACATCTACCCGTACCTGGC
TaGlu2:RP	GACACCACGAGCTTCACGTTG
TaGAPDH:FP	TCACCACCGACTACATGACC
TaGAPDH:RP	ACAGCAACCTCCTTCTCACC

#### *Fusarium* head blight disease progression

Wheat plants were grown in the greenhouse as described previously to quantify *Fusarium* head blight progression on wheat heads. When the inflorescence was in mid-anthesis (growth stage 65; [30]), wheat heads were sprayed with 2 mL of 2 × 10^5^ conidia mL^-1^ *F*. *graminearum* strain DAOM180378 (control plants were sprayed with Tween20) using a hand-held sprayer, as per the protocol by Perochon et al. [23]. To promote infection, heads were covered with plastic bags post-inoculation for four days to increase the humidity [23]. The infection was evaluated and scored visually at ten days post-inoculation. This experiment comprised two independent trials: each trial contained 20 heads (10 plants) per treatment and pots were arranged in a randomised design. At harvest (growth stage 91; [30]), heads were harvested, and the grain was used for mycotoxin analysis.

#### Mycotoxin analysis

Wheat heads collected from the head blight quantification assay experiment was ground and powder was stored at -20°C to quantify the concentration of four mycotoxins: 1) deoxynivalenol (DON), 2) 15-acetylDON (15ADON), 3) DON-3-glucoside (D3G) and 4) sambucinol (SAM). Samples were prepared following a modified Suylok multi-mycotoxin method [31]. Briefly, 1 mL of extraction solvent consisting of 78:20:2 acetonitrile:water:acetic acid was added to 200 mg of ground sample. Samples were vortexed for 30 seconds, followed by sonication for 30 minutes and shaking at 1,400 rpm on a Thermomixer for 30 minutes. Samples were spun down for 10 minutes at 10,000 rpm and a 300 μL aliquot was removed and mixed with 300 μL of LC-MS grade water (Optima Grade, Fisher Scientific, NJ, USA). The solutions were then syringe filtered using a 0.45 μm PTFE filter (Chromspec Inc., ON, Canada) into a 2 mL amber HPLC vial for LC-MS/MS analysis.

Data were collected according to the protocol described by Renaud et al., using a Q-Exactive^TM^ Quadrupole Orbitrap mass spectrometer (Thermo Scientific) coupled to an Agilent 1290 ultra-high-performance liquid chromatography (UHPLC) system [32]. Compounds were resolved using a Zorbax Eclipse Plus RRHD C18 column (2.1× 50 mm, 1.8 μm; Agilent Technologies, CA, USA) at 35°C [32]. The mobile phase comprised of water with 0.1% formic acid (A), and acetonitrile with 0.1% formic acid (B) (Optima grade, Fisher Scientific, NJ, USA). The C18 gradient consisted of 0% B for 30 seconds before increasing to 55% over 1.2 minutes, held isocratically for 2.3 minutes and increased to 100% over 1 minute. Mobile phase B was then held at 100% for 2 minutes before returning to 0% over 30 seconds. The following conditions were used for both positive and negative atmospheric pressure chemical ionization (APCI), as per the protocol by Renaud et al.: discharge current, 3.5 μA; capillary temperature, 300°C; sheath gas, 32.00 units; auxiliary gas, 10.00 units; vaporizer temperature, 250°C; S-Lens RF level, 50 [32]. All analytes were analysed by MS/MS in positive ionization mode except D3G, which was monitored in negative mode as a formate adduct (Table 3). The MS/MS method used an automatic gain control (AGC) of 3 × 10^6^, maximum injection time (max IT) of 64 ms and isolation window of 1.2 m/z. Quantification was accomplished using Thermo Xcalibur^™^ software: a processing method with ICIS peak integration algorithm consisting of 5 smoothing points, baseline window of 70 and 3 ppm mass accuracy.

**Table 3:** Tandem mass spectrometry (MS/MS) information of four analytes of interest: DON, 15ADON, SAM and D3G.

Analyte	Retention time (min)	Ion type	Precursor (*m/z*)	Normalized collision energy	Quantifier/Qualifier (*m/z*)
DON	2.22	[M+H]^+^	297.1	27	203.1064/231.1010
15ADON	2.63	[M+H]^+^	339.1	26	137.0592/231.1006
SAM	2.84	[M+H]^+^	267.2	37	123.0807/95.0860
D3G	2.16	[M+FA]^-^	503.2	25	427.1607/247.0972

### Statistical analysis

Data were analysed through one-way analysis of variance (ANOVA) using RStudio Version (1.1.463) (RStudio In., Boston, MA). Treatment means were separated by the Duncan test (agricolae package) (*p* < 0.05). The results are presented using bar graphs, where bars represent standard error of the mean (SE). Means showing significant differences are represented by differing letters.

## Results

### LSE and chitosan induce resistance of wheat against *F*. *graminearum* infection–leaf detachment assay

**Fig 1:**
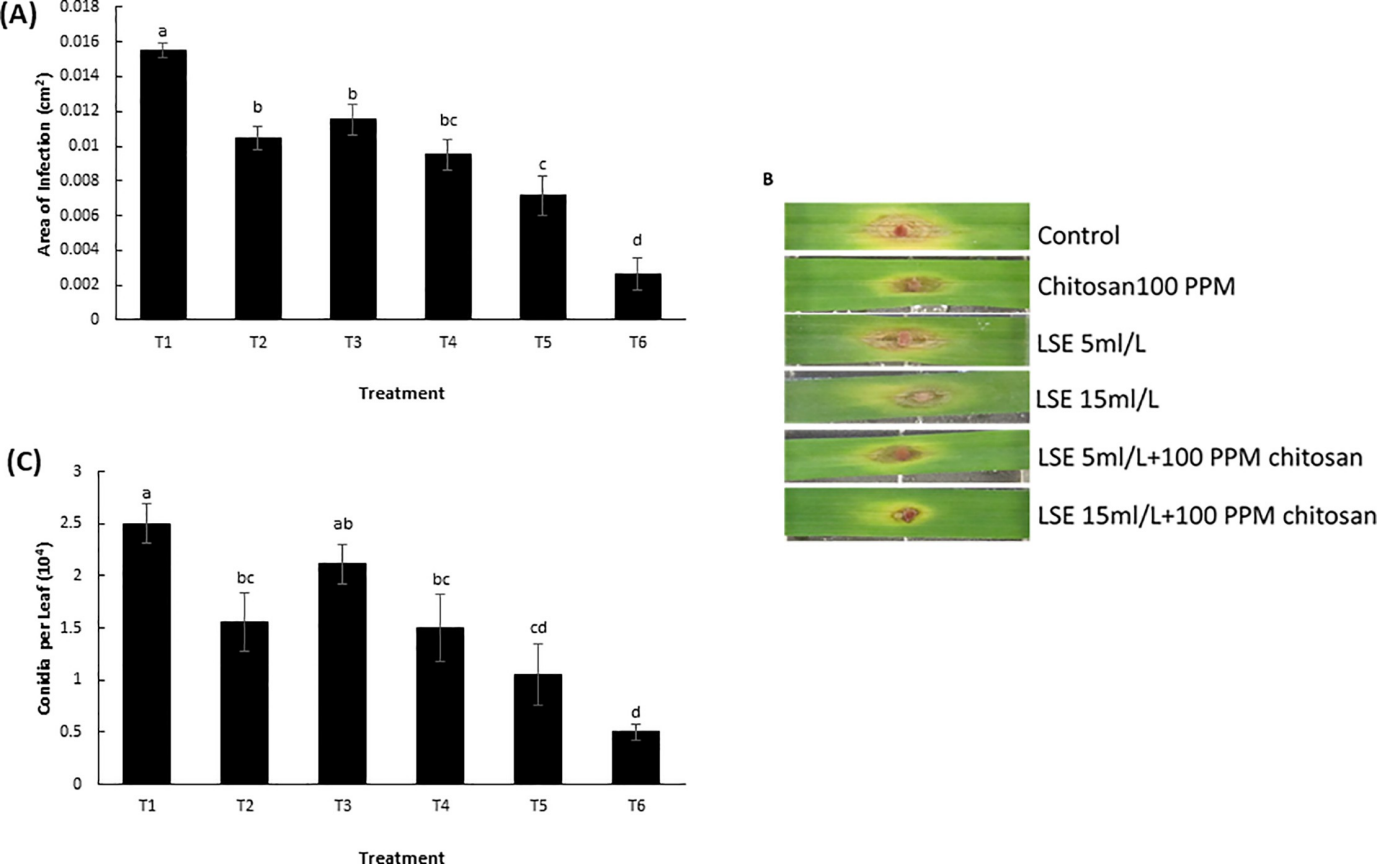
Effect of *Ascophyllum nodosum* extract (LSE) and chitosan on wheat leaf resistance to *F*. *graminearum*. (A) Mean area of infection (cm^2^) on wheat leaves 4 days post-inoculation with *F*. *graminearum* spores. (B) Phenotype of diseased leaf segments. (C) Mean number of conidia (10^4^) produced per leaf. Treatments: T1, Tween 20 (0.02%) (Control); T2, chitosan (100 ppm); T3, LSE (5 mL L^-1^); T4, LSE (15 mL L^-1^); T5, LSE 5 mL L^-1^) + chitosan (100 ppm); T6, LSE (15 mL L^-1^) + chitosan (100 ppm). Different letters indicate significant difference at *p* < 0.05. Bars indicate standard error.

Leaf detachment assay was used to evaluate the effect of LSE, chitosan and its combination on infection and sporulation of *F*. *graminearum* (DAOM180378) on wheat leaf. The area of infection of *F*. *graminearum* on leaf segments were significantly reduced (T2 –T6) compared to the control (T1) (Fig 1A and 1B). The combination of LSE (15 mL L^-1^) and chitosan (T6) significantly reduced the area of infection (80%) on the leaf segments compared to the individual treatments of LSE or chitosan, showing a synergistic effect of the combination. There was a significant reduction in the number of conidia produced per leaf in all treatments compared to the control, except T3 (Fig 1C). Again, the strongest effect was observed in the combination treatment containing LSE (15 mL L^-1^) and chitosan (T6), exhibiting a reduction of 84% in the number of conidia produced when compared to the control treatment. These results suggest that the combination of LSE and chitosan enhance plant resistance to *Fusarium* colonization and reduce spore production.

### Inhibition of mycelial growth of *F*. *graminearum–in vitro* antifungal assay

**Fig 2:**
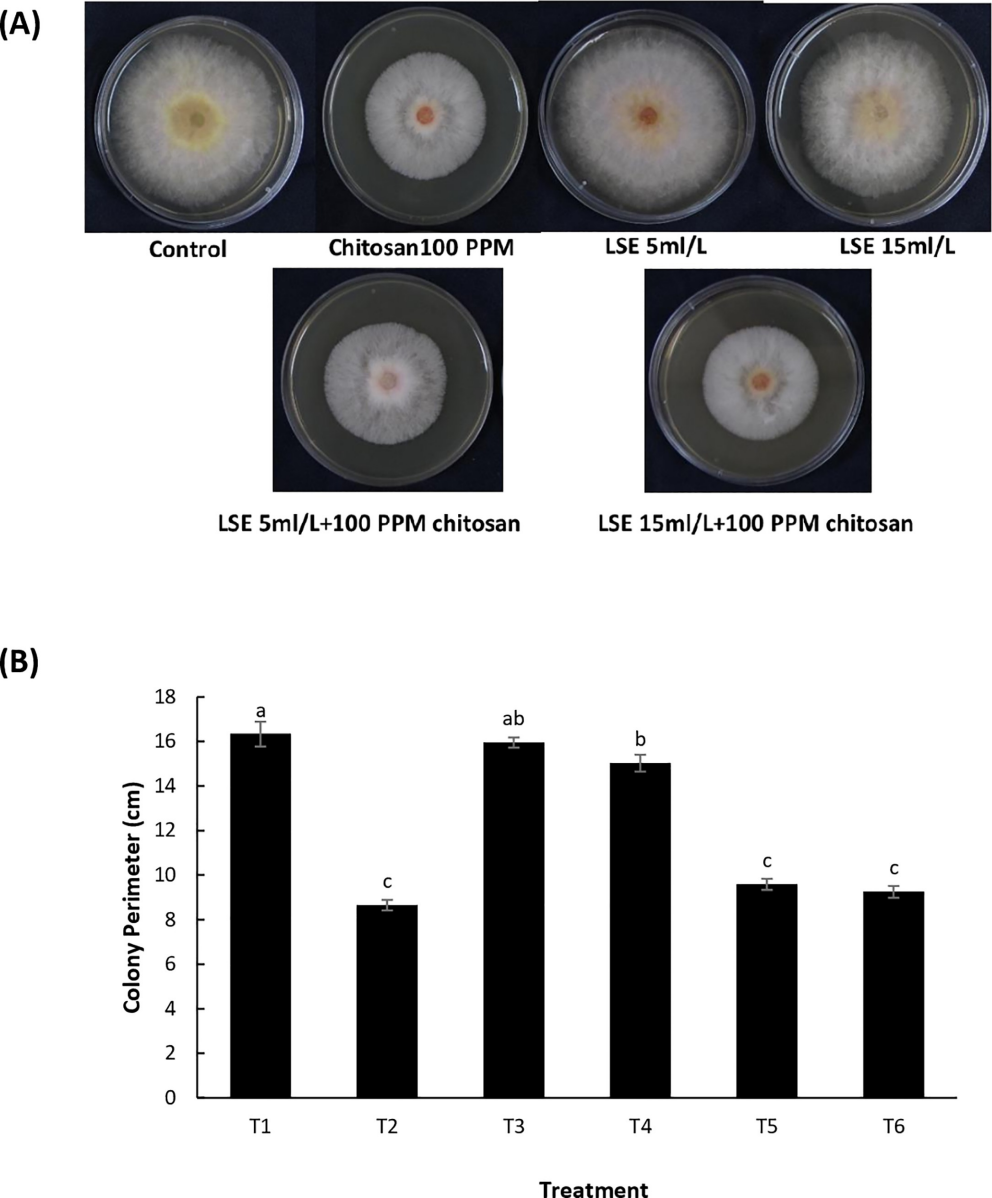
Inhibition of mycelial growth of *F*. *graminearum–in vitro* antifungal assay. (A) Mycelial growth of *Fusarium graminearum* on PDA plates amended with *Ascophyllum nodosum* extract (LSE) and chitosan. (B) Mean colony perimeter of *F*. *graminearum* on PDA plates amended with various concentrations of LSE and chitosan. Treatments: T1, Tween 20 (0.02%) (Control); T2, chitosan (100 ppm); T3, LSE (5 mL L^-1^); T4, LSE (15 mL L^-1^); T5, LSE 5 mL L^-1^) + chitosan (100 ppm); T6, LSE (15 mL L^-1^) + chitosan (100 ppm). Different letters indicate significant difference at *p* < 0.05. Bars indicate standard error.

The effect of LSE and chitosan, individually and in combination, on *F*. *graminearum* mycelial growth was evaluated. LSE and chitosan individually and in combination (T2, T5 and T6) significantly inhibited mycelial growth as compared to the control (Fig 2). There was also a significant reduction of mycelial growth in T4 (15 mL L^-1^ LSE) compared to the control. The combination of LSE and chitosan (T5 and T6) did not statistically increase the inhibitory effect compared to chitosan alone (T2), therefore there was no synergistic effect of LSE and chitosan on mycelial growth.

### Biochemical analysis of defense responsive enzymes in wheat

#### Phenylalanine ammonia-lyase (PAL) activity

**Fig 3:**
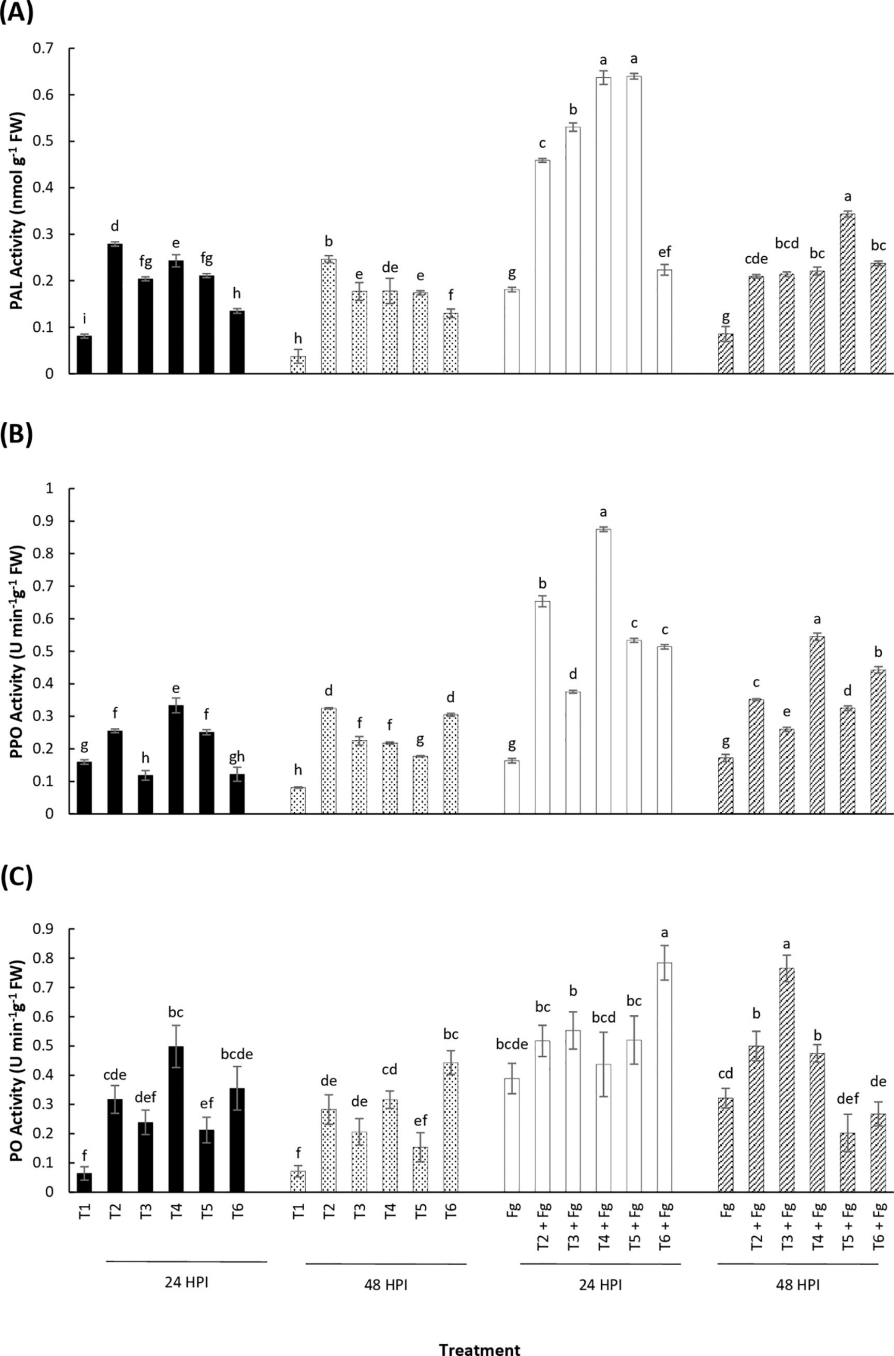
Biochemical analysis of defense responsive enzymes in wheat. Mean activity of plant defense enzymes (A) PAL, (B) PO and (C) PPO in wheat seedlings in response to *Fusarium* infection. Two leaf stage seedlings were drenched with 5 mL of different combinations of LSE and chitosan. Samples were collected for 24 and 48 hours-post pathogen inoculation (HPI). Treatments: T1, Tween 20 (0.02%) (Control); T2, chitosan (100 ppm); T3, LSE (5 mL L^-1^); T4, LSE (15 mL L^-1^); T5, LSE 5 mL L^-1^) + chitosan (100 ppm); T6, LSE (15 mL L^-1^) + chitosan (100 ppm). Different letters indicate significant difference at *p* < 0.05 within each time point. Bars indicate standard error.

The treatment of wheat seedlings with LSE and chitosan, individually or in combination elevated PAL activity, but the response observed was not dose dependent (Fig 3A). PAL activity was also detected in uninoculated and untreated seedlings (control treatments). The greatest PAL activity occurred in treated plants after 24 hours of inoculation with *Fusarium*. Both uninoculated and inoculated plants treated with LSE and chitosan had greater PAL activity than the controls at 24 hours. The chitosan treatment (T2) exhibited greater PAL activity than all other treatments and the control in uninoculated plants after 24 hours, but only showed greater activity than the control in the inoculated plants. Conversely, the combination treatment T5 (LSE 5 ml L^-1^ + chitosan) had a significantly greater PAL activity than the other combination treatment T6 (LSE 15 mL L^-1^ + chitosan) in both uninoculated and inoculated plants after 24 hours. A similar trend can be observed in both uninoculated and inoculated plants treated with LSE and chitosan compared to the controls at 48 hours.

#### Polyphenol oxidase (PPO) activity

Twenty-four hours post-treatment and inoculation, the PPO activity was significantly greater in the inoculated plants compared with the uninoculated plants (Fig 3B). The maximum PPO activity was observed in the inoculated plants treated with T4 (LSE 15 mL L^-1^). The same treatment showed the highest PPO activity in the uninoculated plants as well. After 48 hours, the maximum PPO activity was observed in T4 for inoculated plants, but in T2 and T6 for uninoculated plants. These results show that under the presence of *Fusarium*, the highest PPO activity is observed in plants treated with a greater concentration of LSE (15 mL L^-1^) and that activity is sustained over 48 hours post-inoculation.

#### Peroxidase (PO) activity

Peroxidase activity similarly increased after treatment with various concentrations of LSE and chitosan individually and in combination. Twenty-four hours post-inoculation, PO activity was highest in combination treatment T6 of the inoculated plants compared with other treatments (Fig 3C). The PO activity after 48 hours was highest in plants treated with T3 and inoculated. From the data, it appears that there is a non-significant trend between the combination treatments: at all time points, treatment T6 (LSE 15 mL L^-1^ + chitosan) is greater than the other combination treatment, T5 (LSE 5 ml L^-1^ + chitosan), but the relationship is only statistically significant at 48 hours uninoculated and 24 hours post-inoculation.

### Induction of transcription of defense response genes in wheat in response to *F*. *graminearum*

**Fig 4:**
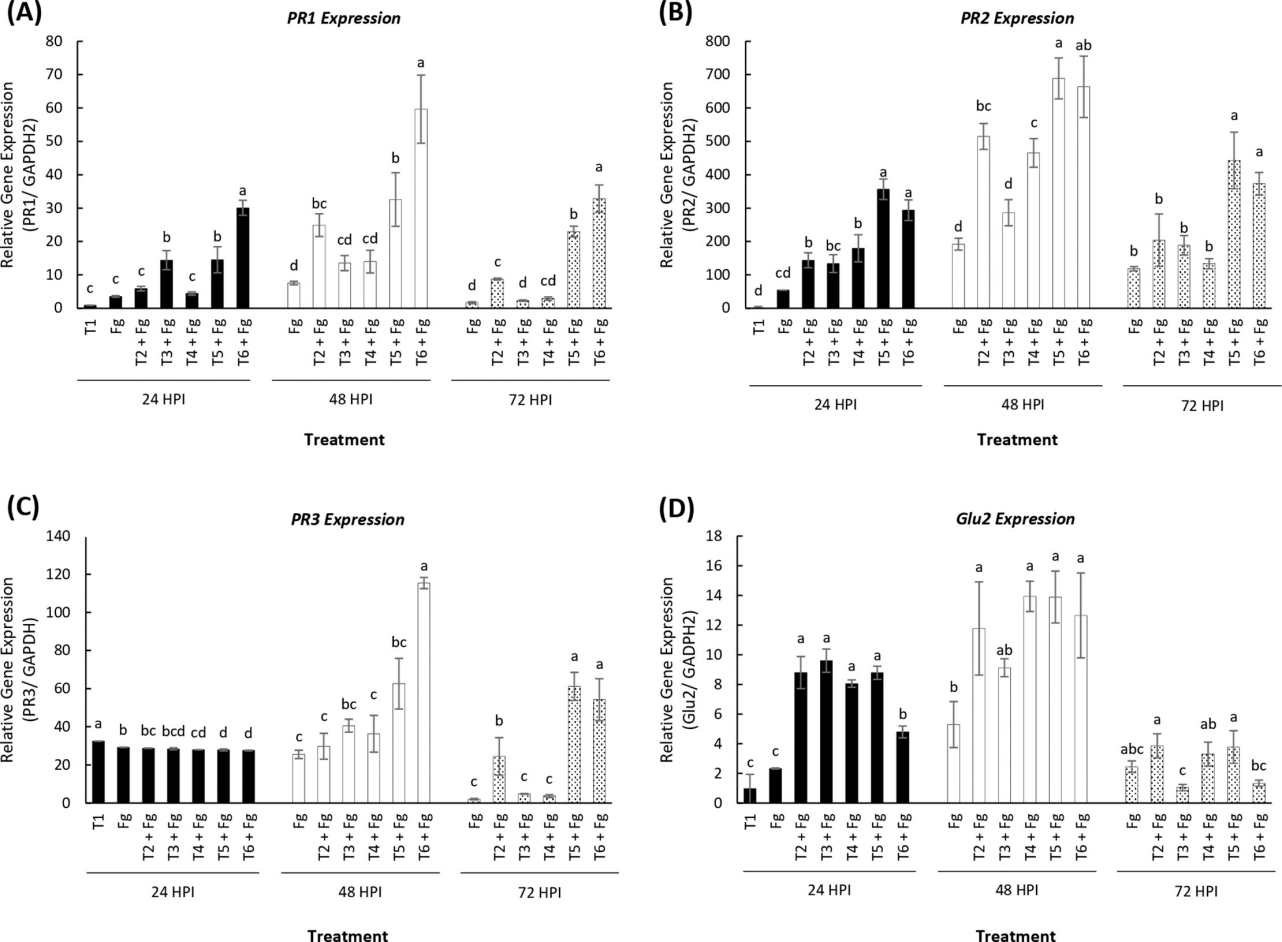
Induction of transcription of defense response genes in wheat in response to *F*. *graminearum*. Mean relative abundance of (A) *TaPR1*, (B) *TaPR2*, (C) *TaPR3* and (D) *TaGlu2* transcripts in wheat seedlings in response to *Fusarium* infection. Two leaf stage seedlings were drenched with 5 mL of treatment. Samples were collected 24, 48 and 72 hours post-pathogen inoculation (HPI). The relative gene expression was calculated using ΔΔCt method using GAPDH as the endogenous reference control. Treatments: T1, Tween 20 (0.02%) (Control); T2, chitosan (100 ppm); T3, LSE (5 mL L^-1^); T4, LSE (15 mL L^-1^); T5, LSE 5 mL L^-1^) + chitosan (100 ppm); T6, LSE (15 mL L^-1^) + chitosan (100 ppm). Different letters indicate significant difference at *p* < 0.05 within each time point. Bars indicate standard error.

The effect of LSE and chitosan in activating defense response genes *TaPR1*, *TaPR2*, *TaPR3* and *TaGlu2* in wheat seedlings was studied. The pathogen response gene *TaPR1* was up-regulated upon *Fusarium* infection in all treatments. Twenty-four hours post-inoculation, *TaPR1* was significantly up-regulated in three treatments (T3, T5 and T6) compared to control (Fig 4A). Forty-eight hours post-inoculation, treatments T2, T5 and T6 showed a significant up-regulation of *TaPR1* compared to the *Fusarium* control. The overall greatest up-regulation of *TaPR1* was observed after 48 hours of inoculation. Seventy-two hours post-inoculation, treatments T2, T5 and T6 were significantly up-regulated compared to the *Fusarium* control.

*TaPR2*, which encodes for β,1–3 glucanase, was significantly up-regulated at 24 hours post-inoculation in all treatments (Fig 4B). Forty-eight hours post-inoculation, all treatments except T3 were significantly up-regulated compared to the *Fusarium* control. Similar to *TaPR1*, the overall greatest up-regulation of *TaPR2* occurred 48 hours post-inoculation. and reached a maximum at 48 hours in the combination treatment of LSE and chitosan (T5 and T6) LSE (Fig 4B). Seventy-two hours post-inoculation, only the combination treatments (T5 and T6) were significantly upregulated compared to the *Fusarium* control. At all time points, the expression of *TaPR2* was significantly greater in both combination treatments (T5 and T6) than the individual treatments.

The defense response gene *TaPR3* that encodes for chitinase showed an interesting pattern over time post-inoculation. Twenty-four hours post-inoculation, there is very little difference between treatments compared to the *Fusarium* control, although the combination treatments (T5 and T6) showed a significantly lower up-regulation than both controls (Fig 4C). However, 48 hours post-inoculation, treatment T5 and 72 hours post-inoculation, both combination treatments (T5 and T6) showed a significantly higher up-regulation of *TaPR3* compared to the *Fusarium* control.

*TaGlu2*, which encodes for β -(1,3; 1,4) glucanase is a key enzyme that degrades the linkages between chitin molecules in the fungal cell wall. All treatments showed a significantly greater up-regulation of *TaGlu2* compared to both controls 24 hours post-inoculation (Fig 4D). The up-regulation of all treatments except for T3 was significantly greater than the *Fusarium* control 48 hours post-inoculation, with no significant difference between treatments at 72 hours.

### Induction of plant resistance to *Fusarium* head blight (FHB)

**Fig 5:**
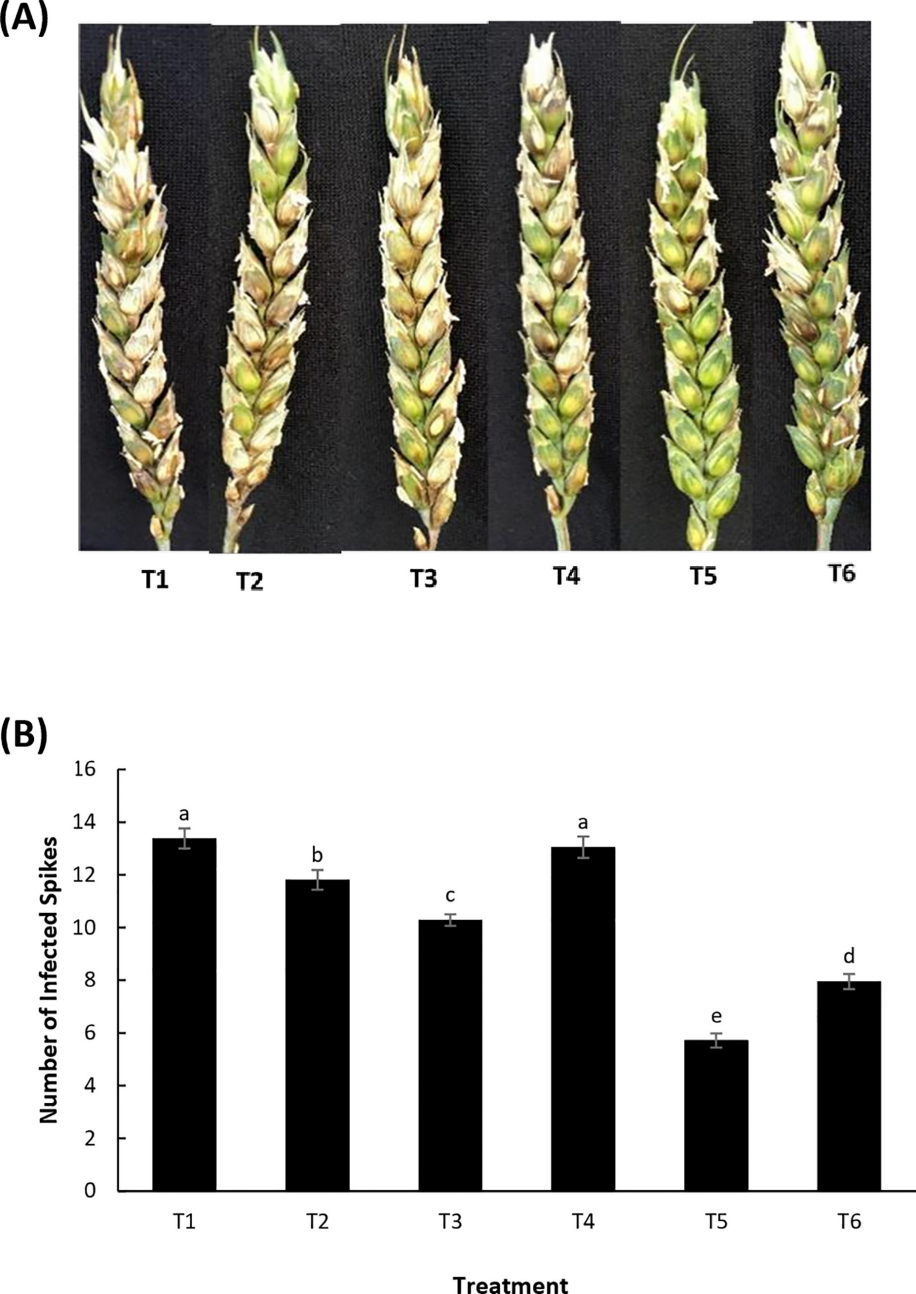
Enhancement of plant resistance to *Fusarium* head blight (FHB). (A) Visualization of *F*. *graminearum* infection in wheat treated with LSE and chitosan 10 days post-inoculation. (B) Mean number of infected spikes per wheat head. Treatments: T1, Tween 20 (0.02%) (Control); T2, chitosan (100 ppm); T3, LSE (5 mL L^-1^); T4, LSE (15 mL L^-1^); T5, LSE 5 mL L^-1^) + chitosan (100 ppm); T6, LSE (15 mL L^-1^) + chitosan (100 ppm). Different letters indicate significant difference at *p* < 0.05. Bars indicate standard error.

The effect of LSE and chitosan on the development of FHB was assessed by comparing the development of disease progression on wheat heads. Plants drenched with LSE and chitosan in combination showed the greatest reduction in *Fusarium* infection when compared to all other treatments (Fig 5B) as well as a reduction in the number of infected spikes 10 days post-inoculation (Fig 5A). There was a reduction of 53.8 and 38.5% in number of infected spikes in T5 and T6 respectively when compared with the control (T1). Based on these results, it appears that there is a synergistic effect of LSE in combination with chitosan in imparting FHB disease resistance in wheat.

### LSE and chitosan reduce mycotoxin concentration in *F*. *graminearum* infected wheat grains

**Fig 6:**
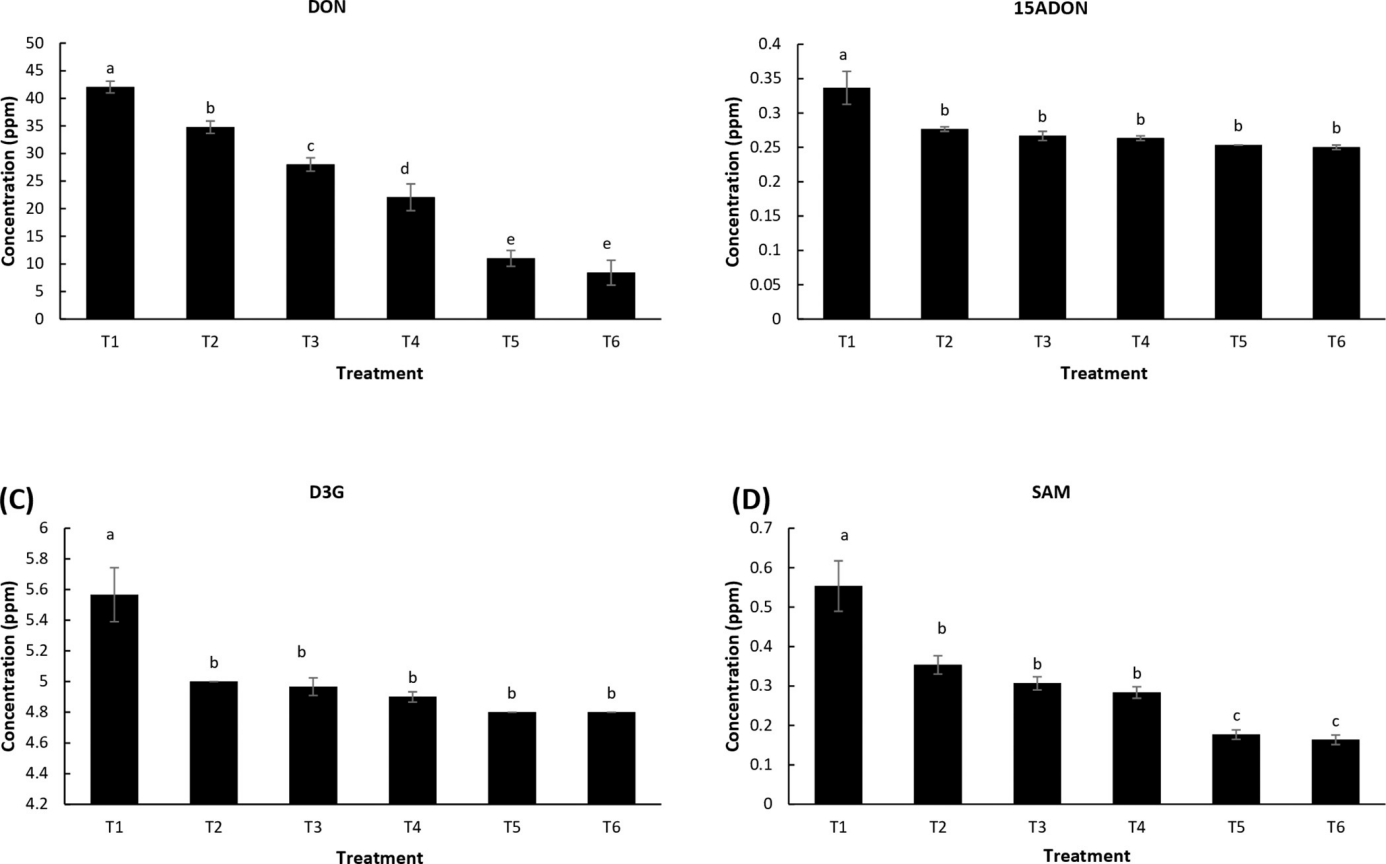
Mycotoxin contamination of wheat kernels upon infection with *F*. *graminearum*. Mean concentration (ppm) of (A) DON, (B) 15ADON, (C) DON3G and (D) SAM in wheat kernels ten days post-pathogen inoculation. Treatments: T1, Tween 20 (0.02%) (Control); T2, chitosan (100 ppm); T3, LSE (5 mL L^-1^); T4, LSE (15 mL L^-1^); T5, LSE 5 mL L^-1^) + chitosan (100 ppm); T6, LSE (15 mL L^-1^) + chitosan (100 ppm). Different letters indicate significant difference at *p* < 0.05. Bars indicate standard error.

Concentration of mycotoxins was analysed in wheat grains harvested in the FHB experiment. In untreated *F*. *graminearum* inoculated plants (control, T1), 42.3 ppm (± 2.5) deoxynivalenol (DON) was detected, whereas all other LSE treatments significantly reduced the concentration of DON in comparison to the control. The greatest reduction of DON was recorded in both combination treatments regardless of the LSE concentration (T5 and T6) (Fig 6A). The acetylated precursor derivative of DON, 15ADON, was also measured in the grains collected from treated wheat heads. When compared with the control, there was a significant reduction of 15ADON in all the treated plants, with no statistical difference between treatments (Fig 6B). The presence of D3G, which is the result of the glycosylation of DON, was also analysed. There was no significant difference of the DON-3-Glucoside concentration between treatments, however, all treatments showed a significantly lower concentration than the control (Fig 6C). The presence of another fusarium derived mycotoxin, SAM, was quantified and all treatments showed a significant reduction in concentration relative to the control treatment (Fig 6D). The highest reduction of SAM was obtained with the combination treatments, T5 and T6 respectively by 70.9%, and 69%.

A minimal dataset of all the experiments are presented in S1 Table.

## Discussion

We have investigated the effect of a plant biostimulant made from *A*. *nodosum* extract (LSE) alone and in combination with chitosan in enhancing wheat plant’s resistance against *F*. *graminearum* (*Fusarium* head blight, FHB disease). Through a detached leaf assay, the combination of LSE and chitosan enhanced plant resistance to *F*. *graminearum* colonization and reduced spore production. Chitosan exhibited a direct antifungal effect on *F*. *graminearum* by reducing radial growth of the pathogen on solid medium. A similar antifungal effect of chitosan was reported for *F*. *oxysporum*, the causal agent of tomato crown and root rot [33] and the amendment of chitosan to solid media inhibited *F*. *solani* f. sp. *phaseoli* growth [34]. More recently, Zachetti et al. [35] reported the addition of chitosan to wheat and maize grains showed a reduction of *F*. *graminearum* growth and mycotoxin contamination. In this experiment, when LSE was combined with chitosan, the antifungal effect was greater than the two compounds used separately.

Greenhouse-grown wheat plants treated with LSE and chitosan exhibited less FHB symptoms and reduced mycotoxin concentration in wheat grains. Khan et al. [12]reported that a chitosan treatment showed a significant reduction of seedling blight symptoms caused by *F*. *culmorum* in wheat and barley by 53 and 91%, respectively. A commercial formulation of *A*. *nodosum* extract, Stimplex^™^, showed a significant reduction in disease incidence of *Alternaria cucumerinum*, *Didymella applanata*, *F*. *oxysporum* and *Botrytis cinerea* in greenhouse-grown cucumbers [36]. Our results show that the application of LSE alone (T3, 5 mL L^-1^) and LSE in combination with chitosan (T5 & T6) resulted in fewer infected spikes of FHB in the greenhouse.

The reduction in FHB symptoms corresponds with the reduction in mycotoxin contamination in wheat grains. It has been reported that the incidence of diseased kernels is directly correlated with mycotoxin load produced by *F*. *graminearum* [37]. Elicitor treatments similarly reduced mycotoxin presence in maize [38,39]. In the present study, mycotoxin analysis showed reduced content of DON, 15ADON and SAM in the wheat kernels, which correlates with diseased spikes in FHB studies. One study suggests the role of cinnamic acid derivatives, such as sinapic, caffeic, p-coumaric, chlorogenic and ferulic acids as the inhibitors of mycotoxin production by *F*. *graminearum* and *F*. *culmorum* [40]. These results support our observations of increased PAL activity: it is hypothesized that because PAL is the first enzyme involved in phenolics synthesis, PAL activity may reduce the mycotoxin production in plants treated with LSE and chitosan.

The LSE and chitosan treatments increased the abundance of pathogenesis-related (PR) protein transcripts as well as plant defense response enzymes such as PAL, PPO and PO, therefore influencing the physiology of the wheat plant. LSE contains various complex polysaccharides (e.g. fucans and alginates) that can act as elicitors of plant defense mechanisms [41,42]. Seaweed extract polysaccharides are reported to stimulate plant defense responses and protect plants against a wide range of microbial pathogens [5,42]. For example, the effects of Stimplex^™^, an *A*. *nodosum* extract formulation, reduced the incidence of *Fusarium* root rot disease on greenhouse-grown cucumbers [36]. The elicitor chitosan has also been shown to induce plant defense and it possesses antimicrobial activity against a wide range of fungi and bacteria [9]. Adding chitosan to a bacterial bio-control formulation increased the efficacy of the formulation against *Fusarium* wilt in tomato [33].

In the current study, we observed an upregulation of various plant defense enzymes in response to LSE and chitosan treatments. The combination of the two further increased enzyme activity compared to the individual treatments. Chitosan alone acts as an elicitor molecule for various defense pathways in plants, so the increased disease resistance of wheat treated with a combination of LSE and chitosan could be attributed to the elicitor activity of compounds present in LSE.

Plant genes encoding pathogenesis-related proteins, β,1–3 glucanases and chitinases are induced upon pathogen attack [43]. Similarly, in this study, LSE and chitosan upregulated genes encoding PR proteins and chitinases. A similar response was reported for *Medicago truncatula* treated with *Ulva* extract which activated plant defense pathways, further enhancing *M*. *truncatula* resistance to *Colletotrichum trifolii* infection [44]. Elicitor molecules present in seaweed extract activate the salicylic acid (SA) and the jasmonic acid (JA) pathways, both plant defense hormone pathways [4,43]. Oligogalacturonides present in LSE can be mobile and induce systemic resistance (ISR) by activating various plant defense pathways and proteinase inhibitors under *in vivo* conditions [44,45].

A foliar spray of chitosan induced defense responses in tomato plants and contributed to resistance of early blight disease cause by *Alternaria solani* [46]. Applying chitosan to celery plants resulted in a 20-fold increase in chitinase activity, a 2-fold increase in β,1–3 glucanase activity and a delay in the onset of symptoms caused by *F*. *oxysporum* [47]. Similarly, we have observed increase PAL, PPO and PO enzyme activity in wheat seedlings infected with *Fusarium* spores. Twenty-four hours post-inoculation, these defense enzymes were induced in all treatments.

Host response is a primary and important trait that reduces *Fusarium* head blight. Modulating plant resistance is one of the most well-documented strategies to reduce FHB disease severity caused by *Fusarium* [48]. Numerous studies reported the induction of plant defense enzymes by a wide range of elicitor molecules [49,50]. In the present study, the combination of LSE and chitosan showed an increased activity of PAL, PPO and PO similar to the results of Jayaraman et al. [36] in which the application of the *A*. *nodosum* extract prolonged enzymatic activity in cucumber leaves up to 72 hours. In another study, the same authours showed a foliar spray of *A*. *nodosum* extract improved resistance against a fungal foliar disease in carrots [51]. Further support for our observations can be found in the activation of LOX and PAL enzymes following a chitosan spray on grape leaves [52].

In addition to the antifungal properties of chitosan, several antioxidants and secondary metabolites of cereals can modulate the production of mycotoxins by various fungal pathogens. Foliar sprays of chitosan induced chitinase, β-1,3-glucanase, and lipoxygenase defense enzyme activities in potato and tomato infected by *Phytophthora infestans* or nematodes [50]. The application of *Ascophyllum* extract reduced *Alternaria* and *Botrytis* foliar blight disease incidence levels in greenhouse-grown carrots, which also showed an elevated expression of pathogenesis-related protein I (*PR-1*), chitinase, lipid transfer protein (*Ltp*), phenylalanine ammonia lyase (*PAL*), chalcone synthase, non-expressing pathogenesis-related protein (*NPR-1*) and pathogenesis-related protein 5 (*PR-5*) [51]. In the present study, we have observed an enhanced expression of PR1, chitinase and β-1,3-glucanase in wheat drenched with chitosan in combination with LSE and challenged with *Fusarium* species.

The expression of plant defense responsive genes might contribute to the resistance of wheat to *Fusarium* in leaf detachment assays and FHB studies under greenhouse conditions. The activation of defense response transcripts and defense enzymes is one of the mechanisms by which LSE and chitosan contribute to the resistance of wheat to *Fusarium*. The high activation of defense enzymes in LSE and chitosan treatments might inhibit the spread of *Fusarium* in wheat heads and in leaf detachment assays. The high activity of enzymes and transcripts in the combination treatments might be attributed to the combined effect of LSE and chitosan in activating defense pathways. Antioxidants modulate the fungal growth and production of mycotoxin production by *Fusarium* species.

With growing concerns about the use of synthetic chemicals in controlling *Fusarium* and the implementation of stringent regulations of chemical pesticides in various countries, there is urgency to find an alternative solution to manage plant diseases. Induction of plant systemic resistance is an effective and attractive approach to combat pathogens. Fortifying LSE with the elicitor chitosan increased the effectiveness of LSE in enhancing resistance against *Fusarium* and FHB. Induction of defense response genes and increased activity of defense response enzymes are possible mechanisms behind enhanced host resistance. With the absence of FHB-resistant cultivars and a ban on effective chemical fungicides, there is a high potential for the implementation of the combination of LSE and chitosan in the management of FHB. Further, a reduction in mycotoxin production under the combined formulation of LSE and chitosan can potentially increase the quality of wheat grains for both animal and human consumption. The improvement of plant health using LSE and chitosan combinations needs to be further evaluated under field environmental conditions.

## Supporting information

S1 Table The supporting information is presented in MS Excel file contains minimal data set of all experiments. Each sheet contains data of one experiment, the title of experiment is presented in each of the sheets.
(XLSX)
